# Changes seen on computed tomography of the chest in mildly symptomatic adult patients with sickle cell disease*

**DOI:** 10.1590/0100-3984.2015.0111

**Published:** 2016

**Authors:** Ursula David Alves, Agnaldo José Lopes, Maria Christina Paixão Maioli, Andrea Ribeiro Soares, Pedro Lopes de Melo, Roberto Mogami

**Affiliations:** 1 MD, Radiologist at the Hospital Universitário Pedro Ernesto (HUPE), Student in the Graduate Program in Medical Sciences at the Universidade do Estado do Rio de Janeiro (UERJ), Rio de Janeiro, RJ, Brazil.; 2 PhD, Adjunct Professor of Pulmonology at the Universidade do Estado do Rio de Janeiro (UERJ), Rio de Janeiro, RJ, Brazil.; 3 PhD, Adjunct Professor of Hematology at the Universidade do Estado do Rio de Janeiro (UERJ), Rio de Janeiro, RJ, Brazil.; 4 PhD, Associate Professor, Head of the Biomedical Instrumentation Laboratory, Head of the Laboratory for Clinical and Experimental Research in Vascular Biology, Universidade do Estado do Rio de Janeiro (UERJ), Rio de Janeiro, RJ, Brazil.; 5 PhD, Adjunct Professor of Radiology at the Universidade do Estado do Rio de Janeiro (UERJ), Head of the Radiology Department of the Hospital Universitário Pedro Ernesto (HUPE), Rio de Janeiro, RJ, Brazil.

**Keywords:** Anemia, sickle cell, Lung diseases/etiology, Tomography, X-ray computed

## Abstract

**Objective:**

To describe and quantify the main changes seen on computed tomography of the chest in mildly symptomatic adult patients with sickle cell disease, as well as to evaluate the radiologist accuracy in determining the type of hemoglobinopathy.

**Materials and Methods:**

A prospective study involving 44 adult patients with sickle cell disease who underwent inspiration and expiration computed tomography of the chest. The frequency of tomography findings and the extent of involvement are reported. We also calculated radiologist accuracy in determining the type of hemoglobinopathy by analyzing the pulmonary alterations and morphology of the spleen.

**Results:**

The changes found on computed tomography scans, in descending order of frequency, were as follows: fibrotic opacities (81.8%); mosaic attenuation (56.8%); architectural distortion (31.8%); cardiomegaly (25.0%); lobar volume reduction (18.2%); and increased caliber of peripheral pulmonary arteries (9.1%). For most of the findings, the involvement was considered mild, five or fewer lung segments being affected. The accuracy in determining the type of hemoglobinopathy (HbSS group versus not HbSS group) was 72.7%.

**Conclusion:**

In adult patients with sickle cell disease, the main tomography findings reflect fibrotic changes. In addition, computed tomography can be helpful in differentiating among hemoglobinopathies.

## Abstract (pt)

**Objetivo:**

Descrever e quantificar as principais alterações na tomografia computadorizada do tórax em pacientes adultos oligossintomáticos com doença falciforme e, secundariamente, avaliar o índice de acerto do radiologista quanto ao tipo de hemoglobinopatia.

**Materiais e Métodos:**

Estudo prospectivo em que 44 pacientes adultos com doença falciforme foram submetidos a tomografia computadorizada do tórax tanto em inspiração como em expiração. Foram descritos a frequência dos achados tomográficos e os graus de acometimento. Por meio da análise das alterações pulmonares e do padrão morfológico do baço, foi calculado o índice de acerto do radiologista quanto ao tipo de hemoglobinopatia.

**Resultados:**

As alterações encontradas nos exames de tomografia computadorizada, em ordem decrescente de frequência, foram: opacidades reticulares (81,8%), padrão de atenuação em mosaico (56,8%), distorção arquitetural (31,8%), cardiomegalia (25%), redução volumétrica lobar (18,2%) e aumento do calibre de ramos periféricos das artérias pulmonares (9,1%). Na maioria dos achados o grau de acometimento foi considerado leve, com até cinco segmentos pulmonares acometidos. O índice de acerto quanto ao tipo de hemoglobinopatia (grupo HbSS *versus* grupo não HbSS) foi 72,7%.

**Conclusão:**

Em pacientes adultos com doença falciforme os principais achados tomográficos refletem alterações fibróticas. Além do mais, a tomografia computadorizada pode ser útil na diferenciação do tipo de hemoglobinopatia.

## INTRODUCTION

Sickle cell disease comprises a group of autosomal recessive hemolytic anemias, all of which involve the presence of hemoglobin S in erythrocytes^(1)^. The most common are homozygous sickle cell disease (HbSS) and thalassemia (HbS), as well as the double heterozygous forms HbSC and HbSD^(1,2)^. Sickle cell disease is one of the most common human hemoglobinopathies, affecting more than 30 million people^(1,2)^. It is estimated that, in Brazil, the disease occurs in 3000 live births per year and that approximately 7.2 million people are carriers of the mutated gene^(3)^.

The simple substitution of an amino acid in the beta chain of hemoglobin leads to a complex network of molecular interactions that alter the permeability and stability of erythrocyte membranes, as well as causing endothelial adhesion, vascular occlusion, and severe hemolysis^(4,5)^. The pathogenesis of vascular involvement in sickle cell disease has been attributed primarily to hemolysis caused by a reduction in the bioavailability of nitric oxide, a potent endogenous vasodilator; there is also a reduction in the activity of the arginine/nitric oxide synthase pathway. Those changes lead to significant vasoconstriction^(6,7)^. In sickle cell disease, there is also thrombin production caused by a chronic inflammatory response, which activates the coagulation cascade and abnormally increases phosphatidylserine exposure on the surface of erythrocytes^(8)^.

Sickle cell disease has a chronic clinical course that is punctuated by acute episodes. A vaso-occlusive, or sickling, crisis is characterized by the occurrence of acute pain in the bones, chest, or abdomen, being most common in patients with HbSS^(9)^. Acute chest syndrome manifests as chest pain, hypoxemia, and pulmonary opacities. The syndrome also occurs in patients with non-HbSS disease, although the clinical and radiological manifestations are milder than those occurring in patients with HbSS^(9,10)^. The spleen is also a place of constant sickling. In adulthood, most HbSS patients become functionally asplenic because of recurrent infarcts of the microvasculature. In non-HbSS patients, there are no splenic infarctions, and, unlike HbSS patients, non-HbSS patients generally show splenomegaly with occasional crises of splenic sequestration^(9,11)^.

The organ most often affected by sickle cell disease is the lung^(12,13)^. However, in the few existing studies on the topic, the exact incidence, prevalence, and natural history of chronic lung disease associated with sickle cell disease are not well established; nor is there a consensus regarding the best methods by which it can be diagnosed^(4,14-19)^. It has been suggested that 4% of patients with sickle cell disease develop lung involvement^(4,14)^, which leads to recurrent episodes of infarction and infection, resulting in intense oxidative stress and vascular remodeling with proliferation of the muscle layer and fibrosis^(14,15)^. There are also pulmonary sequelae presenting as recurrent episodes of acute chest syndrome^(14)^, which is the greatest risk factor for the development of chronic lung disease^(13)^.

Pulmonary complications account for 20-30% of all deaths in adults with sickle cell disease^(16)^. Programs of neonatal screening have led to the early diagnosis of various inherited diseases, including sickle cell disease, resulting in a considerable reduction in infant mortality rates and better survival. Consequently, abnormalities in pulmonary function test results, as well as interstitial lung disease identified by computed tomography (CT), have been described with everincreasing frequency^(14,17,18)^. In a prospective study of 29 patients who had experienced one to more than 10 (median, six) previous episodes of acute chest syndrome, interstitial abnormalities were seen on a CT scan of the chest in 12 (41%), the severity and extent of those abnormalities being associated with the number of episodes of the syndrome^(17)^.

In patients with sickle cell disease, tomographic findings include mosaic attenuation, loss of lung volume, prominent central vessels, ground-glass opacities, irregular linear opacities, traction bronchiectasis, interlobular septal thickening, and nodules^(18,19)^. A finding of fibrosis on CT is associated with restrictive lung disease on pulmonary function tests, which shows the importance of CT in the detection of respiratory abnormalities in this patient population^(18)^.

There have been few studies of the changes seen on chest CT scans in mildly symptomatic patients with chronic sickle cell disease. Therefore, it is important for to be aware knowledge of the forms of presentation of the main pulmonary alterations identified by radiologists, in order to improve understanding of the pathophysiology of the disease, as well as to facilitate the stratification by severity and the selection of treatment strategies. The primary objective of this study was to describe and quantify the principal changes seen on chest CT scans of mildly symptomatic patients with sickle cell disease. A secondary objective was to evaluate radiologist accuracy in determining the type of hemoglobinopathy.

## MATERIALS AND METHODS

We studied 44 mildly symptomatic adult patients with sickle cell disease who were followed at our institution. We included patients who were ≥ 18 years of age, regardless of the treatment applied, with no acute inflammatory comorbidities at the time of evaluation. Patients with a history of pulmonary tuberculosis were excluded, as were those with collagen diseases, those infected with HIV, those with a history of smoking, and those with heart valve disease or lung disease unrelated to sickle cell disease. The study was approved by the local research ethics committee, and all participating patients gave written informed consent.

We acquired CT scans of the chest (including the upper abdomen) using a 64-channel multislice CT scanner (Brilliance 40; Philips Medical Systems, Cleveland, OH, USA). With the patient in the supine position, we acquired images in the axial plane, from the suprasternal notch to the xiphoid process, at maximum inspiration and expiration, using the following technical parameters: 120 kV and 458 mAs (which can vary depending on patient biotype); slice thickness of 2 mm; a pitch of 1; and no gantry tilt. After image acquisition, we performed high-resolution reconstruction with a 512 × 512 matrix and a high-frequency algorithm, using a window with a width of 1200 HU and a mean center of -800 HU. No intravenous contrast was administered during any of the examinations. The tomographic findings were interpreted independently by two radiologists with experience in examinations of the chest (10 years and 4 years, respectively). Disagreements were resolved by consensus.

For each abnormality, we used adapted staging criteria, according to the number of lung segments affected^(20-22)^. The following CT findings were evaluated: reticular opacities, mosaic attenuation, lobar volume reduction, and signs of pulmonary hypertension. In evaluating the CT scans, we used the term "mosaic attenuation" in order to define airways disease as well as vascular occlusive disorders, the latter being implicated in the pathophysiology of sickle cell disease^(23)^.

The degree of involvement seen on CT was graded as follows^(21,22)^: 0 (no lung segments affected); 1 (1-5 lung segments affected); 2 (6-9 lung segments affected); or 3 (more than 9 lung segments affected). The abnormalities were defined in accordance with the criteria established by the Fleischner Society^(24)^ and the Brazilian illustrated consensus terminology of descriptors and fundamental patterns on chest CT scans^(23)^. By analyzing pulmonary abnormalities and the morphological pattern of the spleen (including atrophy, calcifications, and splenomegaly), we also calculated the accuracy of radiologists in determining the type of hemoglobinopathy.

## RESULTS

Of the 44 study participants, 32 were carriers of the homozygous mutation (HbSS), or sickle cell disease, and 12 were carriers of the heterozygous mutation (HbSC or HbSB). Of those 44 patients, 29 were female and 15 were male. The mean age was 54 ± 9.9 years. The mean values for weight, height, and body mass index were 71.5 ± 10.6 kg, 165 ± 20 cm, and 26.3 ± 4.8 kg/m^2^, respectively. Forty patients (90.9%) were being treated with hydroxyurea, although not on a regular basis.

**Figure 1:**
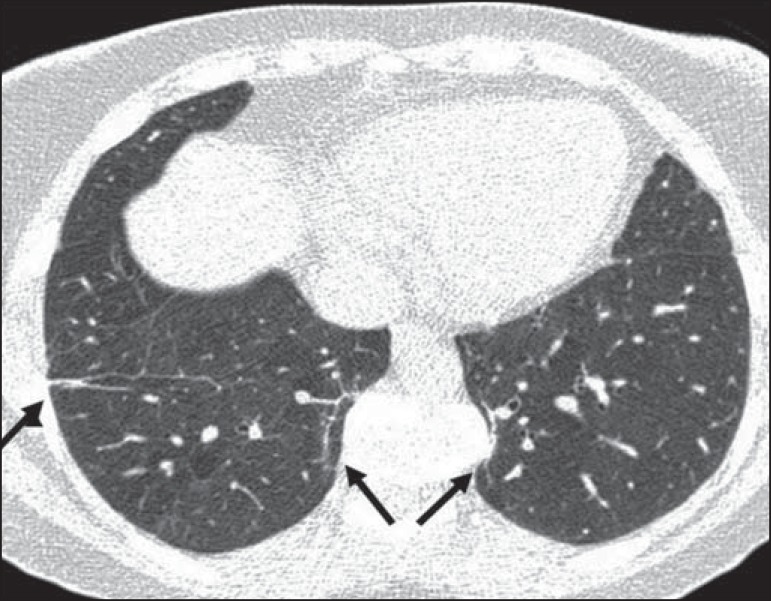
Chest CT scan of a patient with sickle cell disease, showing reticular opacities in the lower lobes (arrows).

**Figure 2:**
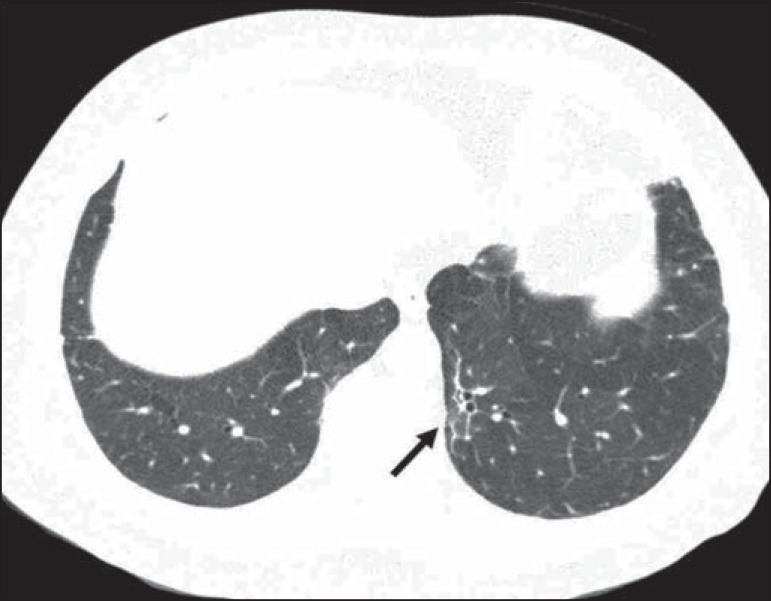
Axial chest CT scan showing mosaic attenuation in the lower lobes. Note the reticular opacity with traction bronchiectasis (arrow).

**Figure 3:**
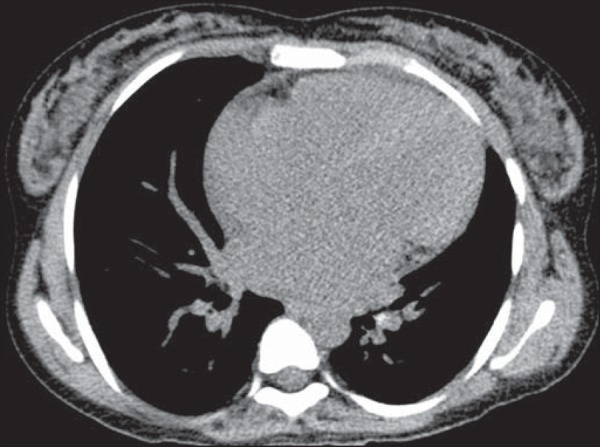
Chest CT scan, with a mediastinal window, of a 23-year-old patient with sickle cell disease, showing cardiomegaly.

**Figure 4:**
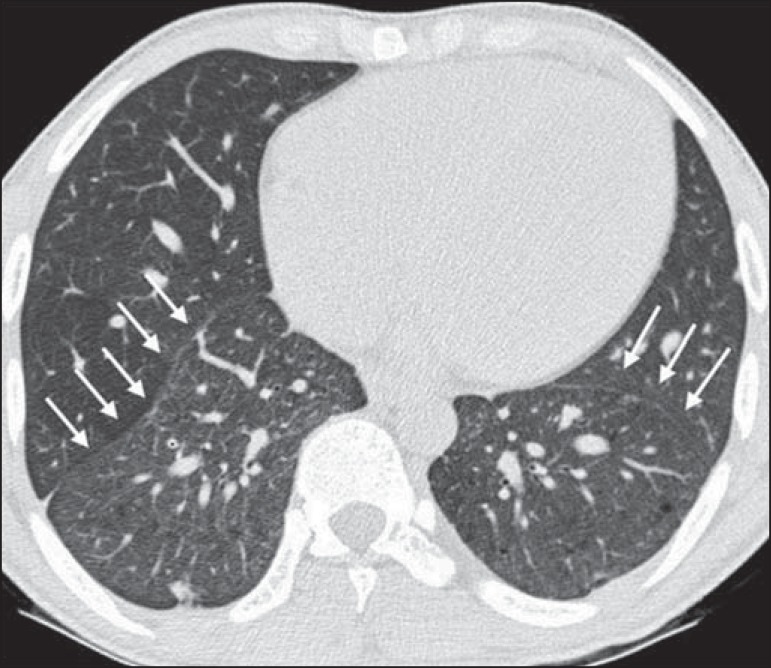
Axial chest CT scan showing significant volume reduction in the lower lobes, especially on the right (arrows).

**Figure 5:**
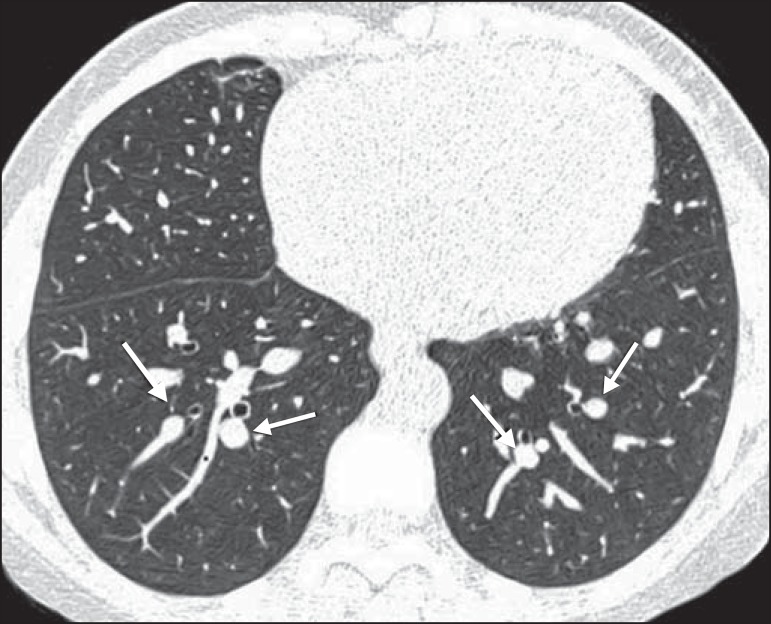
Axial chest CT scan showing increased caliber of the peripheral pulmonary vessels (arrows).

The findings on CT scans, in decreasing order of frequency, were as follows: reticular opacities (Figures 1 and 2), in 81.8% of the patients; mosaic attenuation (Figure 2), in 56.8%; architectural distortion and cardiomegaly (Figure 3), in 31.8% and 25.0%, respectively; lobar volume reduction (Figure 4), in 18.2%; and increased caliber of the peripheral pulmonary arteries (Figure 5), in 9.1%. An increase in the caliber of the pulmonary artery trunk was observed in only one patient.

Among the 36 patients who had reticular opacities, the opacities were classified as grade 1 (1-5 lung segments affected) in 21 (58.3%), as grade 2 (6-9 lung segments affected) in 11 (30.5%), and as grade 3 (more than 9 lung segments affected) in only 4 (11.1%).

Mosaic attenuation, which was identified in 25 patients, was classified as grade 2 (6-9 lung segments affected) in 14 (56.0%) of the patients affected and occurred in the lower lobes in 23 (92.0%). Lobar volume reduction was observed in 8 patients and was classified as grade 1 (1-5 lung segments affected) in 5 (62.5%), the lower lobes being affected in 7 (87.5%). These findings and their frequencies are shown in Table 1.

**Table 1:** Grading of chest computed tomography findings in the 44 patients evaluated

Computed tomography finding	G0	G1	G2	G3
Reticular opacities	8	21	11	4
Mosaic attenuation	19	9	14	2
Lobar volume reduction	36	5	3	0
Signs of pulmonary hypertension	43	1	0	0

G0, no lung segments affected; G1, 1–5 lung segments affected; G2, 6–9 lung segments affected; G3, more than 9 segments affected.

**Figure 6:**
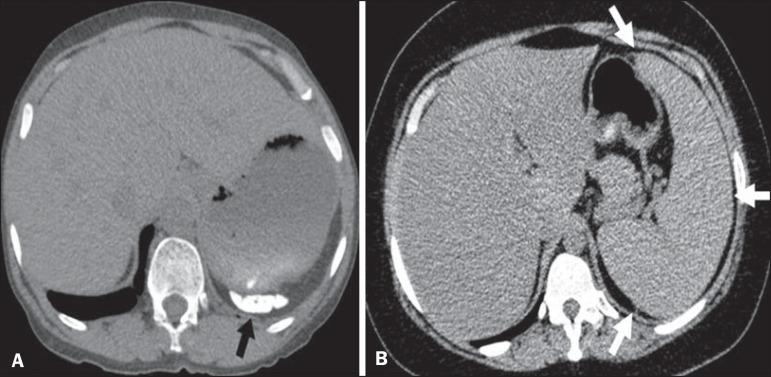
Different patterns of splenic involvement in sickle cell disease. Sickle cell disease (HbSS) shows atrophy and calcification of the spleen (arrow on **A**), whereas non-HbSS sickle cell disease can show splenomegaly (arrows on **B**).

The radiologist success rate in determining the type of hemoglobinopathy (HbSS versus non-HbSS) was 72.7%. The radiologist determined the type of hemoglobinopathy correctly in 32 of the 44 cases evaluated (Figure 6).

## DISCUSSION

Chest CT has been shown to be an important tool for the study of parenchymal lung disease, as has recently been discussed in the radiology literature of Brazil^(25-32)^. Contrary to what has been observed in some studies^(4,14)^, in which findings indicative of chronic lung disease have been reported in only 4% of patients with sickle cell disease, we observed a high frequency of such changes, which were present in 88.6% of the patients evaluated. This can be attributed, at least in part, to the greater longevity of the patients in our sample, which can be attributed to the use of hydroxyurea. Treatment with hydroxyurea elevates levels of fetal hemoglobin and increases the longevity of circulating erythrocytes, thereby minimizing the deleterious effects associated with chronic hemolysis^(18)^. It is of note that all of the patients in our study were ≥ 18 years of age and that most were under treatment with hydroxyurea.

The tomographic findings in the present study are consistent with those described in other studies, including reticular opacities, mosaic attenuation, reduced lung volume, and architectural distortion characterized by irregular linear opacities and traction bronchiectasis^(18,19)^. The most common finding was reticular opacities, which were likely sequelae of previous acute chest crises and pulmonary infarctions. Previous studies have shown that the severity and extent of alterations seen on CT scans correlate directly with the number of episodes of acute chest syndrome^(14,17)^. Another quite common finding of the present study was architectural distortion of the lung parenchyma, which was characterized by extensive reticular opacities accompanied by retraction of the lung parenchyma and traction bronchiectasis.

We observed mosaic attenuation in 56.8% of the cases evaluated. Mosaic attenuation is a nonspecific sign that is indicative of occlusive vascular disease as well as of airway disease^(33,34)^. In most cases, images acquired in expiratory apnea identify individuals with small airways disease as a cause of mosaic attenuation^(34)^. However, such images might not be reliable for distinguishing between small airways disease and small-vessel disease in complex pathophysiological situations in which both the vessels and airways are abnormal, as is the case in patients with sickle cell disease^(35)^. Therefore, in our description of the CT examinations, we chose to use the term "mosaic attenuation", which is more comprehensive than is the term "air trapping", even when the images were acquired in expiratory apnea.

Airway abnormalities have been described in patients with vascular occlusive disease, especially when there is dilation of segmental and subsegmental bronchi in areas affected by chronic occlusive events^(35)^, although there are few data in the literature regarding the mechanism involved. We observed increased caliber of the peripheral pulmonary arteries, a finding that is attributed to the presence of small thrombi within the lumen^(36)^, in only four patients.

Cardiomegaly, which was observed in 25% of the patients evaluated in the present study, has been described in other studies of patients with sickle cell disease as a result of systolic and diastolic ventricular dysfunction, high cardiac output caused by chronic anemia, or pulmonary hypertension^(37)^. The patients included in the present study underwent frequent clinical follow-up evaluations, showing normal echocardiography results or pulmonary artery pressure values at the upper limit of normality (data not shown). The sensitivity and specificity of CT in identifying pulmonary arterial hypertension, by measuring the caliber of the pulmonary trunk, are relatively low^(38)^. That could explain the low proportion of patients with increased pulmonary artery caliber in the present study.

We observed a low incidence of tomographic findings with the maximum grade of involvement, especially grade 3 findings. That can be explained, at least in part, by the close clinical monitoring and early treatment of the patients in our sample, which in turn slows or reduces the effect of chronic hemolysis and the inflammatory cascade in the pulmonary microvasculature. It is noteworthy that, in the present study, the radiologists showed high accuracy in determining the type of hemoglobinopathy, on the basis of the alterations seen in the lungs and in the spleen. To our knowledge, there have been no previous studies testing this hypothesis.

A critical analysis of the results of this study and its limitations is warranted. First, the study had a cross-sectional design and the sample size was relatively small. However, the objective of the study was to evaluate CT findings in sickle cell disease patients with greater longevity, which greatly restricted the number of patients in the sample. Second, the CT findings could have been better defined if we had also studied hemodynamics and pulmonary function. This applies, for example, to mosaic attenuation, a finding that can reflect small airways disease or small vessel disease, which are indistinguishable on CT, particularly in complex pathophysiological situations in which the vessels and the airways are both affected. Despite these limitations, we believe that our results make an important contribution, given that there have been few studies of chest CT findings in patients with sickle cell disease. Further studies, with a longitudinal design and involving larger patient samples, should be performed in order to improve understanding of the pathophysiology of sickle cell disease, promoting early, regular treatment and multidisciplinary follow-up. That could increase survival and reduce the social stigma associated with this chronic disease that provokes significant thoracic alterations.

In conclusion, the results of the present study show that there is high frequency of alterations seen on chest CT in mildly symptomatic patients with sickle cell disease, the most common being fibrotic changes, which probably represent sequelae of acute chest crises or pulmonary infarctions. In addition, CT can be useful in differentiating the type of hemoglobinopathy.
